# Profiling Human-AI Regulatory Support in Technology-Enhanced Interprofessional Education Among Health Professions Students: Person-Centered Exploratory Study

**DOI:** 10.2196/95772

**Published:** 2026-09-10

**Authors:** Fraide A Ganotice Jr, John Ian Wilzon T Dizon, Xiaoai Shen

**Affiliations:** 1 Bau Institute of Medical and Health Sciences Education, Li Ka Shing Faculty of Medicine, The University of Hong Kong, 5/F William MW Mong Block, 21 Sassoon Road, Pokfulam, Hong Kong SAR, Hong Kong, Hong Kong SAR, HKG, China (Hong Kong), 852 3917 9814

**Keywords:** self‑regulated learning, coregulation, generative AI, cluster analysis, interprofessional education, health professions

## Abstract

**Background:**

Technology-enhanced health care interprofessional education (IPE) places high demands on students’ self-regulated learning (SRL) and their ability to work productively with others to prepare them for collaborative practice in health care settings. Yet, little is known about how health professions students perceive and combine their own SRL with coregulation from human and AI-based support in such environments.

**Objective:**

This exploratory and observational study adopted a person-centered approach to identify regulatory profiles based on self-reported SRL and perceived coregulation with near-peer teachers (NPTs) and generative AI (GenAI), and to examine how these profiles were associated with interprofessional learning outcomes.

**Methods:**

Health professions students (N=136) enrolled in a technology-enhanced IPE completed an SRL questionnaire at the beginning of the program. They rated coregulation from NPTs at midprogram and coregulation from GenAI at the end, together with communication, collaboration, and learning satisfaction. A 2-stage exploratory clustering approach using SRL and coregulation scores as indicators was used to identify distinct regulatory profiles. Profile differences in interprofessional communication, collaboration, and learning satisfaction were examined using independent samples 2-tailed *t* tests.

**Results:**

A 2-profile solution provided the best fit to the data. The positive human-NPT–GenAI regulation profile (51/136, 37.5%) was characterized by moderately high SRL, high coregulation with NPTs, and above-average coregulation with GenAI. The negative human-NPT–GenAI regulation profile (85/136, 62.5%) showed the opposite pattern, with moderately low SRL, low coregulation with NPTs, and below-average coregulation with GenAI. Students in the positive profile reported significantly higher interprofessional communication (*P*=.005) and collaboration (*P*=.006) and greater learning satisfaction (*P*<.001) than those in the negative profile, with medium to medium-large effect sizes.

**Conclusions:**

These findings provide person-centered evidence that self-regulation and coregulation from human and AI agents form distinct perceived regulatory configurations in technology-enhanced IPE and that a richer “regulatory ecology,” combining stronger SRL with greater perceived coregulation from near-peer and GenAI support, was associated with more favorable interprofessional outcomes. The study highlights the importance of deliberately designing near-peer teaching and GenAI-supported activities as complementary regulatory scaffolds in health professions education.

## Introduction

### Background

To set the stage for recognizing the importance of self-regulation and coregulation in technology-enhanced interprofessional learning, consider the following scenario. Before a near-peer–led, case-based tutorial, Maria, a health professions student, reads the patient scenario, reviews the learning objectives, and sets personal goals for the session. During the tutorial, she and her peers work with a near-peer teacher (NPT) who helps them clarify priorities, prompts them to explain their reasoning, and suggests strategies for approaching similar cases. Maria uses a checklist to follow the discussion, asks focused questions, and briefly consults a generative AI (GenAI) tool to explore alternative diagnoses and management plans. After the session, she reviews her notes and the GenAI-generated suggestions, identifies any remaining points of confusion, and adjusts her study plan.

This pattern of planning, strategy use, monitoring, and reflection is termed self-regulated learning (SRL): learner’s proactive, cyclical control over their cognition, motivation, behavior, and environment to achieve learning goals [1,2]. Yet Maria does not regulate alone. Her NPT and the GenAI tool each serve as coregulatory agents that temporarily scaffold her goal-setting, monitoring, and strategy adaptation [3]. Although interprofessional education (IPE) is widely regarded as essential for preparing students to collaborate across disciplinary boundaries [4], it remains unclear how students combine their own SRL with coregulation from these human and AI-based supports, or whether different combinations carry implications for learning outcomes.

In health professions education, higher levels of SRL have been linked not only to better academic performance but also to more effective clinical reasoning and stronger orientations toward lifelong learning [5-7]. Technology-enhanced learning environments, including blended formats, simulation platforms, and GenAI tools, are now increasingly central to health professions curricula [8-10], and research has shown that students’ SRL is a critical determinant of success in such settings [11,12]. Theoretical advances have extended individual SRL models to encompass coregulation, the transitory process by which a more knowledgeable agent guides a learner’s planning, monitoring, or reflection, and have situated both constructs within collaborative learning environments [3,13]. Near-peer teaching has demonstrated cognitive and affective benefits for health professions learners [4,14], and GenAI tools now offer an always-available source of guidance that can explain concepts, suggest plans, and critique answers on demand [9,15].

Despite these advances, 3 gaps persist. First, most SRL research uses variable-centered methods that estimate average effects across samples, potentially obscuring distinct subgroups of learners who configure their regulatory resources differently [16,17]; person-centered methods have rarely been applied in IPE settings (confer [18,19]). Second, coregulation has been examined almost exclusively in relation to human agents; how students engage with GenAI as a coregulatory support remains empirically unexamined. Third, no study has jointly profiled SRL alongside coregulation from both a human and an AI-based source to determine whether distinct configurations exist and whether they relate to educational outcomes.

To address these gaps, the present study adopted a person-centered approach to identify distinct regulatory profiles among health professions students enrolled in a technology-enhanced IPE program that incorporated both near-peer teaching and GenAI tools. Profiles were derived from students’ self-reported SRL and their perceived coregulation from NPTs and GenAI across the program, and were then compared on interprofessional communication, collaboration, and learning satisfaction. In doing so, this study extends the near-peer teaching literature by reframing NPTs as coregulatory agents within an SRL framework and advances the emerging GenAI literature by examining GenAI not in isolation but as part of a coordinated human-AI regulatory ecology in technology-enhanced IPE.

### SRL-Coregulation Framework

We draw on multilevel regulation of learning perspectives [3,13], which distinguish between regulation by the individual learner (self-regulation) and regulation that is supported by others or tools (coregulation). Although these perspectives also describe socially shared regulation of learning (group-level regulation enacted jointly by a team), socially shared regulation is not the focus of the present study and was not measured.

Coregulation describes episodes in which another person or tool deliberately scaffolds a learner’s regulation, for example, by clarifying goals, proposing strategies, prompting monitoring, or supporting emotion regulation. In this study, we focus on NPTs and GenAI as 2 complementary coregulatory agents within the same regulatory cycle: near-peers provide human scaffolding in small-group-learning, while GenAI offers on-demand, tool-based guidance.

This SRL-coregulation perspective is grounded in Winne and Hadwin [20] cyclical model of SRL, in which learners move through the phases of task definition, goal setting and planning, enactment, and adaptation. Across these phases, regulation can target cognition (eg, strategy choice), motivation (eg, sustaining effort), emotions (eg, managing frustration), and behavior (eg, time management). Coregulation operates within the same cycle: effective coregulation provides temporary scaffolds that can strengthen learners’ SRL over time.

Within this framework, baseline SRL is conceptualized as a relatively stable individual resource that students bring into the IPE program. Coregulation from NPTs (mid of the program) and from GenAI (end of the program) represents external regulatory supports embedded in the same instructional context. This lens allows these to be treated as components of a single regulatory system, and the resulting profiles (eg, high SRL or high coregulation; low SRL or low coregulation) to be interpreted as different configurations of individual and distributed regulatory capacity.

### From Variable-Centered to Person-Centered Regulation

Most empirical work on SRL, coregulation, and technology has adopted variable-centered approaches, correlating average SRL scores or perceived instructional support with outcomes such as grades, satisfaction, or perceived competence [5,7]. These studies show that, on average, stronger SRL and higher-quality support are beneficial. Yet, variable-centered analyses assume that the same relationships hold for all students and cannot reveal whether there are qualitatively distinct subgroups who combine SRL and coregulation in different ways, for example, highly self-regulated students who seldom seek help vs low SRL students who rely heavily on external scaffolding.

Person-centered approaches, such as cluster analysis and latent profile analysis, offer a complementary lens. They group individuals based on patterns of scores across multiple variables, thereby identifying naturally occurring profiles rather than relying solely on averages. In motivational and emotional research, person-centered analyses have revealed profiles such as “high positive or low negative emotions” or “high autonomous or low controlled motivation,” which are differentially associated with engagement and achievement [16,17,21]. In digital learning contexts, students’ SRL strategies and behaviors in technology-rich environments also cluster into distinct profiles linked to persistence and achievement [11,22]. However, very few studies have used cluster analysis to examine regulatory profiles that explicitly combine SRL with coregulation, and, to our knowledge, none has derived SRL-coregulation profiles where coregulation is provided by both human NPTs and GenAI within an authentic educational setting.

### The Present Study

This study had 2 primary goals. First, we sought to identify regulatory profiles of health professions students in a technology-enhanced IPE, based on their baseline SRL, perceived coregulation from NPTs, and perceived coregulation from GenAI. Because this is one of the earliest person-centered investigations that jointly model human and AI-based coregulation within an SRL-coregulation framework, we did not specify a hypothesis about the exact number of profiles. Conceptually, however, extreme combinations can be described in terms of more vs less SRL and more versus less coregulation (eg, “positive” vs “negative” regulatory ecologies).

Importantly, the present study was exploratory and observational. Although the data were collected across 3 time points, students were not randomly assigned to different regulatory supports, and the focal constructs were measured through self-report. Therefore, the study does not test whether NPT or GenAI support causes improvements in communication, collaboration, or satisfaction. Rather, it examines whether distinct perceived self-regulation and coregulation profiles can be identified and whether membership in these profiles is associated with study outcomes.

The first research question (RQ) is as follows: what self-regulation and coregulation profiles emerge among health professions students in a technology-enhanced IPE program, based on SRL and coregulation with NPTs and GenAI?

Second, we examined how profile membership related to interprofessional learning outcomes, operationalized as students’ end-of-program ratings of communication and collaboration, and of their learning satisfaction. Prior work has shown that, on average, higher SRL and higher quality instructional support are associated with better collaborative competence and satisfaction. Extending this to a person-centered context, we expected the more “resource-rich” regulatory profiles to show more adaptive outcomes.

The second RQ is as follows: do self-regulation and coregulation profiles differ in interprofessional communication, collaboration, and learning satisfaction at the end of the IPE program?

The hypotheses are as follows:

- H2.1: students in a profile characterized by relatively higher SRL and stronger coregulation with both NPTs and GenAI would report higher interprofessional communication and collaboration than those in a profile characterized by lower SRL and weaker coregulation.
- H2.2: students in the higher regulation profile would also report greater learning satisfaction than those in the lower regulation profile.

### Significance

Theoretically, this study extends SRL-coregulation perspectives on multilevel regulation of learning to a technology-enhanced IPE context by conceptualizing NPTs and GenAI as complementary coregulatory agents within a single distributed regulatory system [3,13]. For the educational technology community, this study illustrates how internal SRL and external, digitally mediated supports can be modeled jointly as regulatory ecologies, aligning with ongoing work on learner profiling, learning analytics, and AI-supported regulation of learning (eg, [11,12,22]). The study also has relevance beyond IPE. Many contemporary educational settings, including team-based learning, project-based learning, clinical simulation, online collaborative learning, and AI-supported higher education, require learners to coordinate their own regulation with support from peers, facilitators, digital tools, and, increasingly, GenAI systems.

Practically, it provides an evidence-based way to identify distinct regulatory profiles among health professions students and to link these profiles to interprofessional communication, collaboration, and learning satisfaction, thereby informing the design of targeted supports for vulnerable learners and the deliberate use of near-peer teaching and GenAI as regulatory scaffolds. Methodologically, it introduces a person-centered, multiwave-design using a 2-stage cluster analysis with pre-post outcome measurement and, to our knowledge, is among the first to integrate SRL with coregulation from both human near-peers and GenAI within one IPE program.

## Methods

### Participants and Context

Participants (N=136) were health professions students from 2 universities in Hong Kong and 1 in Mainland China, all enrolled in a compulsory, cross-institutional IPE stroke rehabilitation simulation hosted by a government-subsidized university in Hong Kong (institution blinded for review). The analytic sample comprised only those with complete data at all 3 time points and included students from Chinese medicine (n=4), medicine (n=13), nursing (n=46), pharmacy (n=18), social work (n=10), physiotherapy (n=30), and speech and hearing sciences (n=15), who worked in mixed-profession teams on technology-enhanced, case-based learning activities. Of these, 80 (58.8%) were female participants, 55 (40.4%) were male participants and one’s sex was not reported (0.7%); most were undergraduates in year 2 (n=61, 44.9%), year 3 (n=35, 25.7%), year 4 (n=30, 22.1%), or year 5 (n=1, 0.7%), with an additional 9 (6.6%) master’s-level physiotherapy students.

### Ethical Considerations

The study formed part of a broader evaluation of the IPE program and was reviewed and approved by the Human Research Ethics Committee of the University of Hong Kong (approval number: EA260174). Participation was voluntary and had no bearing on students’ course grades or progression. Data were collected and stored in accordance with institutional data protection policies and the Declaration of Helsinki. All students in the targeted cohorts were invited to participate, and only those who provided informed consent and completed all 3 survey waves were included in the analyses.

### IPE Program

**Figure 1.**
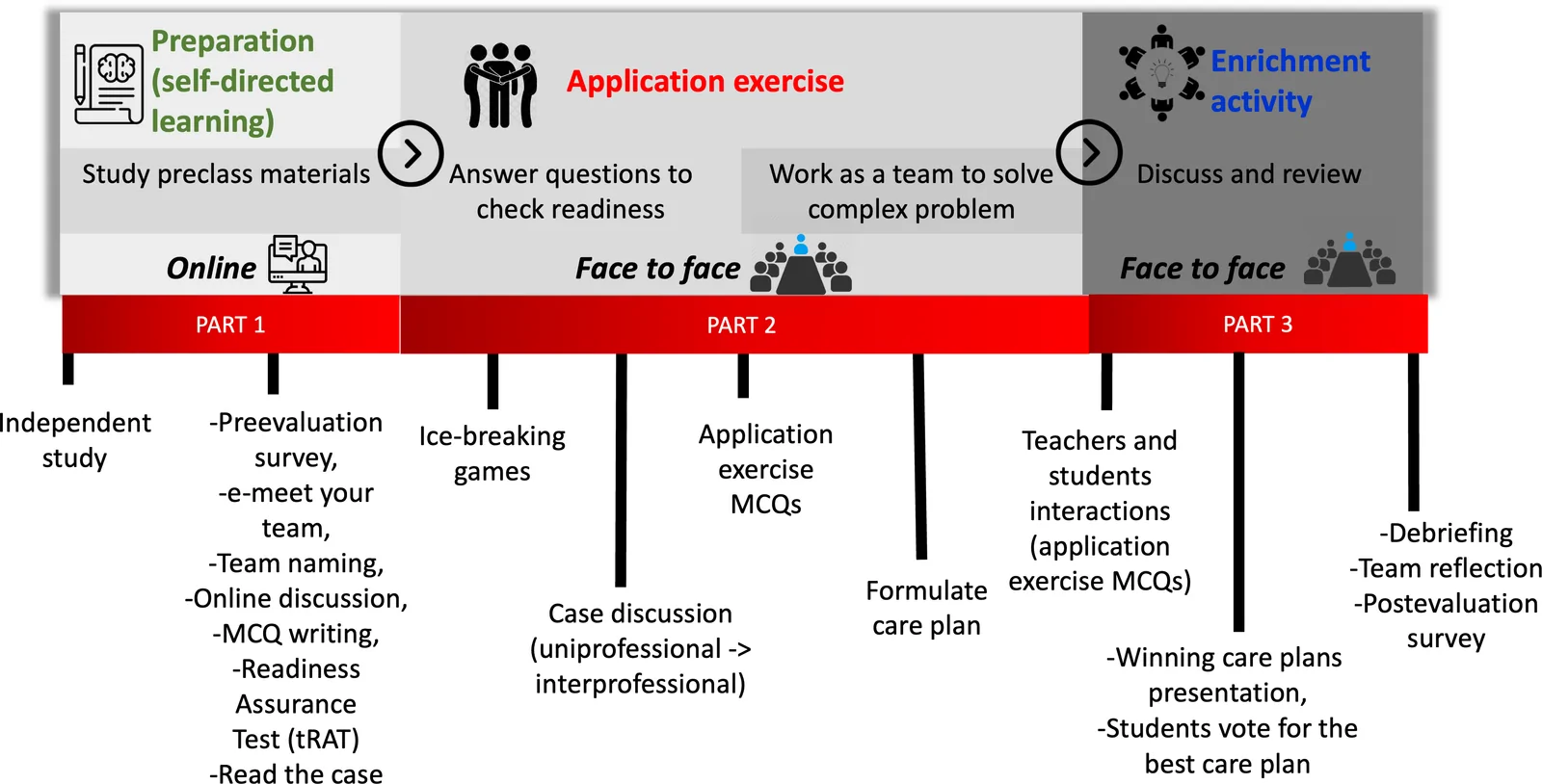
Learning activities in the interprofessional education (IPE) program. MCQ: multiple choice question.

The IPE program centered on a stroke rehabilitation simulation designed to support interprofessional communication, role clarification, collaborative care planning, and team-based decision-making. The program ran over 2 months and included 3 parts (Figure 1): part 1 was preparation, part 2 was application exercise, and part 3 was enrichment activity. These activities were intended to help students practice communication and collaboration in a simulated but authentic interprofessional context. Students were allocated to mixed-profession teams so that each team included learners from multiple health and social care disciplines. Across the program, teams worked through a shared patient case, discussed discipline-specific and interprofessional priorities, developed collaborative management plans, and reflected on team processes. The technology-enhanced format included online survey administration, hybrid team activities, and structured opportunities to use GenAI during selected learning tasks.

### Procedure

Data were collected at 3 time points aligned with key phases of the IPE program. At the beginning of the program (time 1; before part 1), before any IPE activities, students completed an online survey assessing baseline SRL and demographic or background information. At midprogram (time 2; at the end of part 2), after a month-long block of near-peer-facilitated IPE activities delivered in a hybrid format (both in-person and online), students completed a second survey. During this block, interprofessional teams engaged in core IPE tasks such as team-based care planning and team readiness assurance tests designed to socialize students into interprofessional collaborative practice, particularly by strengthening interprofessional communication and teamwork. Each team was randomly assigned a trained NPT. Each NPT was a senior student (eg, undergraduate year 4 or year 5) who had completed or was completing structured preparation in the Peer Teaching Certificate Programme, which required NPTs to view 4 training videos, participate in peer-teaching activities, and submit a final reflective essay to obtain the certificate. Their role was facilitative rather than primarily didactic: they helped teams clarify task goals, prompted students to explain their reasoning, encouraged participation across professions, monitored group progress, and supported reflection on communication and collaboration. In the time-2 survey, students rated the extent to which their NPT coregulated their learning in these activities.

At the end of the program (time 3; at the end of part 3), following IPE activities that explicitly incorporated GenAI tools, students completed a third survey assessing perceived coregulation from GenAI, interprofessional collaborative competencies, and overall learning satisfaction.

GenAI was introduced as a learning support rather than as an authoritative clinical decision-maker. A university-provided GenAI platform, integrating ChatGPT (OpenAI), DeepSeek (Hangzhou DeepSeek Artificial Intelligence Co, Ltd), and Gemini, was available during these activities, although students were also permitted to use other GenAI tools, where possible, to select tools that they considered capable and appropriate for the learning task. This decision was intended to reflect authentic student use of GenAI in contemporary learning environments, where learners often interact with a range of publicly available or personally accessible tools.

Students were encouraged to use GenAI to clarify concepts in the stroke-rehabilitation case, compare possible care planning options, check the completeness of team reasoning, and support posttask reflection. Students also received general guidance on the responsible use of GenAI, including how to frame clear prompts, critically evaluate GenAI-generated responses, and compare outputs with course materials, professional knowledge, and team reasoning. However, no fixed or standardized prompt templates were provided. They were also reminded to evaluate GenAI outputs critically and not to treat them as authoritative clinical advice. Accordingly, the GenAI measure in this study captures students’ perceived regulatory support from self-selected GenAI tools, rather than exposure to a standardized platform. Actual GenAI use was not objectively tracked; therefore, we did not collect usage logs, record which tools students used, or measure the frequency, duration, or content of their GenAI interactions.

All 3 surveys were administered online via Qualtrics, took approximately 15 to 20 minutes to complete, and included at least one instructed-response item (for example, “Please select ‘agree’ for this item”) as well as system-logged completion time to enhance data quality. Responses were anonymous, and only participants who provided complete data across all 3 time points were included in the present analyses.

### Measures

#### Regulation Measures

All regulation constructs were assessed with 9 items adapted from the Metacognitive Self-Regulation subscale of the Motivated Strategies for Learning Questionnaire (MSLQ [23]). Wording was modified so that items referred either to students’ self-regulation, by NPTs, or by GenAI. In all cases, responses were given on a 7-point Likert scale (1=“strongly disagree” to 7=“strongly agree”) and averaged, with higher scores indicating stronger self-regulation or coregulation.

#### SRL (Time 1)

SRL at time 1 was measured with 9 items capturing goal setting, strategic planning, monitoring of understanding, and reflection (eg, “I ask myself questions to make sure I know the material I have been studying”). Higher scores indicate stronger SRL. Confirmatory factor analysis indicated an acceptable model fit for this scale: incremental fit index (IFI) of 0.980, goodness of fit index (GFI) of 0.956, normed fit index (NFI) of 0.925, root mean square error of approximation (RMSEA) of 0.050, and standardized root mean square residual (SRMR) of 0.054.

#### Coregulation From NPTs (Time 2)

Perceived coregulation by NPTs at time 2 was assessed with an adapted version of the same item set, reworded so that the NPT was the regulator. Items tapped four domains: (1) task structuring and goal clarification, (2) strategic guidance, (3) monitoring and feedback, and (4) motivational or emotional support (eg, “Our near-peer teacher helped clarify goals for each interprofessional task”). Higher scores reflect stronger perceived coregulation by NPTs. Model fit indices for this scale were IFI of 0.980, GFI of 0.931, NFI of 0.964, RMSEA of 0.096, and SRMR of 0.079.

#### Coregulation From GenAI (Time 3)

Perceived coregulation by GenAI was measured with parallel items in which the regulator was the AI system rather than the NPT. The items covered the same 4 domains: task structuring and goal clarification, strategic guidance, monitoring and feedback, and motivational or emotional support (eg, “The AI tool helped me plan how to approach each interprofessional task”). Higher scores indicate stronger perceived coregulation by GenAI. Model fit indices for this scale were IFI of 0.986, GFI of 0.948, NFI of 0.965, RMSEA of 0.070, and SRMR of 0.036.

#### Communication and Collaboration (Time 3)

Communication and collaboration were measured at time 3 using 2 subscales from the Interprofessional Collaborative Competencies Attainment Survey [24]. The communication subscale captured participants’ self-assessed abilities in facilitating effective interprofessional dialogue, active listening, expressing ideas constructively, and providing feedback (eg, “Actively listen to IP team members’ ideas and concerns”). Responses were given on a 7-point scale (1=“strongly disagree” to 7=“strongly agree”; 5 items) and were averaged; higher scores indicate greater communication competencies. The collaboration subscale assessed participants’ perceived competence in proactively engaging with team members, working cooperatively to enhance care, and learning from interprofessional interactions (eg, “Work effectively with IP team members to enhance care”). Responses were given on a 7-point scale (1=“strongly disagree” to 7=“strongly agree”; 3 items) and were averaged; higher scores indicate greater collaboration competencies.

#### Learning Satisfaction (Time 3)

Postprogram learning satisfaction was measured at time 3 using the Learning Satisfaction Questionnaire [25]. Items captured overall satisfaction with the IPE experience, perceived usefulness for future professional practice, and satisfaction with opportunities to develop interprofessional skills (eg, “Overall, I am satisfied with this interprofessional education programme”). Responses were given on a 5-point scale (1=“strongly disagree” to 5=“strongly agree”; 4 items) and were averaged; higher scores indicate greater satisfaction.

### Demographic and Background Variables (Time 1)

At time 1, students reported their health profession program, year of study, sex, and prior IPE experience (eg, none vs some). These variables were used to describe the sample.

### Data Analysis

Data analyses were conducted in SPSS (version 28; IBM). We first screened the data for missingness, outliers, and assumptions of normality. Cases with missing data on all 3 profile indicators (SRL, near-peer coregulation, and GenAI coregulation) were removed. Additional cluster-solution robustness checks were conducted in R (version 4.5.3; R Core Team [26]) using the cluster package.

We computed descriptive statistics (means and SDs) for all variables and examined internal consistency reliability (Cronbach α) for each multi-item scale. Confirmatory factor analyses were conducted to evaluate the measurement properties of the SRL, coregulation with NPT, and coregulation with GenAI. Model fit was judged using standard indices (eg, IFI, GFI, RMSEA, and SRMR). Pearson correlations among SRL, coregulation indicators, ICCAS scores, satisfaction, and demographic variables were then inspected.

To address the first research question, we conducted a 2-stage exploratory clustering approach, which is appropriate to handle continuous data and ensure statistical robustness [27]. We used time 1 SRL, time 2 coregulation with NPT, and time 3 coregulation with GenAI as continuous profile indicators. Indicators were standardized (as *z* scores) prior to analysis. Initially, to extract the number of clusters from the data, we performed a hierarchical cluster analysis using Ward method and squared Euclidean distance, and we identified 2 clusters based on the resulting dendrogram. Consequently, we ran a k-means cluster analysis to identify the final cluster centers and classify the participant’s cluster membership.

We additionally compared the retained 2-cluster solution with 3-cluster and 4-cluster alternatives using the same standardized profile indicators and k-means clustering procedure. Because no single index is definitive in determining the optimal number of clusters, cluster quality was evaluated using average silhouette values, with Calinski-Harabasz values, cluster sizes, interpretability, and parsimony inspected as supplementary considerations.

To address the second research question, we ran an independent 2-tailed *t* test with 1000 bootstrap samples and examined whether there would be significant differences in participants’ collaboration and communication outcomes and learning satisfaction based on the 2 cluster profiles yielded. A *P* value less than .05 was considered significant, and Cohen *d* of 0.2 (small effect size), 0.5 (medium effect size), and 0.8 (large effect size) were used as reference points for interpretation.

## Results

The descriptive statistics and correlational analyses are presented in Tables 1 and 2. The reliability coefficients (Cronbach α) were acceptable for all variables. Students’ self-regulation was positively associated with coregulation from NPTs (*r*=0.358; *P*<.001), postintervention communication competencies (*r*=0.275; *P*=.001), and postintervention collaboration competencies (*r*=0.230; *P*=.007), but was not significantly related to coregulation from GenAI (*r*=−0.066; *P*=.44) or learning satisfaction (*r*=0.127; *P*=.14). Coregulation from NPTs was positively related to postintervention communication (*r*=0.175; *P*=.04), postintervention collaboration (*r*=0.213; *P*=.01), and learning satisfaction (*r*=0.271; *P*=.001). Similarly, coregulation from GenAI was positively associated with communication (*r*=0.178; *P*=.04), collaboration (*r*=0.175; *P*=.04), and learning satisfaction (*r*=0.353; *P*<.001). Notably, postintervention communication and collaboration competencies were highly intercorrelated (*r*=0.927; *P*<.001), and both were strongly associated with learning satisfaction (communication: *r*=0.624, *P*<.001; collaboration: *r*=0.613, *P*<.001).

**Table 1.** Descriptive statistics (N=136).

Variable	Score, mean (SD; range)	Skewness (SE)	Kurtosis (SE)	Cronbach α
Self-regulation	4.90 (0.71; 1‐7)	0.18 (0.20)	0.95 (0.41)	0.72
NPT^a^ coregulation	5.13 (0.80; 1‐7)	0.38 (0.20)	−0.08 (0.41)	0.73
Gen-AI coregulation	4.44 (0.86; 1‐7)	−0.27 (0.20)	0.42 (0.41)	0.79
Communication	5.32 (0.98; 1‐7)	−0.62 (0.20)	2.16 (0.41)	0.96
Collaboration	5.36 (1.03; 1‐7)	−0.78 (0.20)	1.99 (0.41)	0.94
Learning satisfaction	3.83 (0.65; 1‐5)	−0.21 (0.20)	0.62 (0.41)	0.88

a NPT: near-peer teacher.

**Table 2.** Correlational analyses.

Variable	SR^a^	NPTCR^b^	GenAICR^c^	Communication	Collaboration	LS^d^
SR, *r*	—^e^	.36^f^				
NPTCR, *r*	.36^f^	—				
GenAICR, *r*	−.07	.097	—			
Communication, *r*	.28^g^	.18^h^	.18^h^	—		
Collaboration, *r*	.23^g^	.21^h^	.18^h^	.93^f^	—	
LS, *r*	.13	.27^g^	.35^f^	.62^f^	.61^f^	—

a SR: self-regulation.

b NPTCR: near-peer teacher coregulation.

c GenAICR: generative AI coregulation.

d LS: learning satisfaction.

e Not applicable.

f *P*<.001.

g *P*<.01.

h *P*<.05.

**Figure 2.**
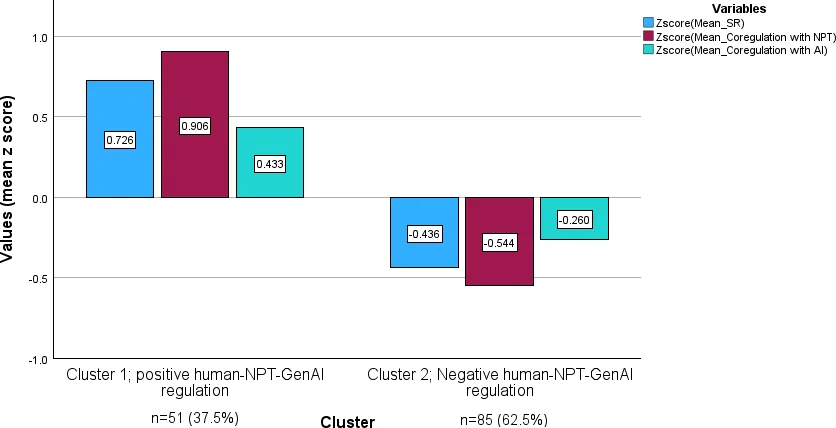
Final cluster centers for self-regulation and coregulation profiles by human–near-peer teacher (NPT)–generative AI (GenAI) regulation cluster (N=136).

Figure 2 depicts the standardized scores for self-regulation and coregulation with NPTs and GenAI across the 2 profiles. The first profile, labeled positive human-NPT–GenAI regulation (cluster 1; 51/136, 37.5% of participants), was characterized by moderately high self-regulation, high coregulation with NPTs, and above-average coregulation with GenAI. Students in this profile, therefore, appeared to combine stronger personal self-regulatory resources with active engagement with both human and AI-based coregulatory scaffolds.

The second profile, labeled negative human-NPT–GenAI regulation (cluster 2; 85/136, 62.5% of participants), showed the opposite pattern. These students reported below-average self-regulation, low coregulation with NPTs, and slightly below-average coregulation with GenAI. This profile thus reflected learners who reported relatively weak internal regulatory resources alongside limited use of both human and AI-based coregulatory supports.

Alternative cluster solutions were compared using the same 3 standardized indicators. Table 3 summarizes the cluster sizes and quality indicators for the 2-cluster solutions, 3-cluster solutions, and 4-cluster solutions. The 2-cluster solution yielded the highest average silhouette value and the strongest Calinski-Harabasz value among the compared solutions. The 3-cluster and 4-cluster alternatives also produced smaller or imbalanced clusters and did not offer a clearer substantive interpretation. Taken together, the quality indicators, interpretability, and parsimony of the profiles supported retaining the 2-profile solution.

**Table 3.** Comparison of alternative cluster solutions.^a^

Solution	Cluster sizes	Average silhouette	Calinski-Harabasz	Interpretation
k=2	85, 51	0.297	60.10	Best quality indicators; retained solution
k=3	75, 51, 10	0.262	43.73	Lower silhouette and Calinski-Harabasz values
k=4	60, 45, 22, 9	0.215	39.61	Lowest silhouette and 1 small cluster

a Higher average silhouette and Calinski-Harabasz values indicate stronger relative support among the compared solutions.

We also explored the distribution of the yielded cluster profiles across the participants’ sex, discipline, and year level. Table 4 shows that regarding sex, female participants were more represented overall (80/136, 58.8%) and were distributed similarly across both clusters (cluster 1 [positive]: 29/51, 56.9%; cluster 2 [negative]: 51/85, 60%), as were male participants (cluster 1: 21/51, 41.2%; cluster 2: 34/85, 40%). Across disciplines, nursing constituted the largest group in both clusters (cluster 1: 16/51, 31.4%; cluster 2: 30/85, 35.3%), followed by physiotherapy (cluster 1: 12/51, 23.5%; cluster 2: 18/85, 21.2%) and pharmacology and pharmacy (cluster 1: 6/51, 11.8%; cluster 2: 12/85, 14.1%). Notably, all Chinese medicine students were classified in the positive regulation group (cluster 1: n=4/51, 7.8%), while speech and hearing sciences students were more concentrated in the negative regulation group (cluster 2: 12/85, 14.1%) relative to the positive regulation group (cluster 1: 3/51, 5.9%). With respect to year level, year 2 students (combining the half-class and new curriculum cohorts, total n=61) were the largest year-level group overall, though year 2 new curriculum students were notably more represented in the negative regulation group (cluster 2: 24/85, 28.2%) than in the positive regulation group (cluster 1: 7/51, 13.7%). Year 4 students were similarly more prevalent in the negative regulation group (cluster 2: 21/85, 24.7%) than the positive regulation group (cluster 1: 9/51, 17.6%). Overall, the distribution of participants across clusters was broadly comparable by sex, discipline, and year level.

**Table 4.** Composition of extracted human–near-peer teacher (NPT)–generative AI (GenAI) regulation clusters by sex, discipline, and grade (year level).

Variable	Cluster 1: positive human-NPT–GenAI regulation (n=51), n (%)	Cluster 2: negative human-NPT–GenAI regulation (n=85), n (%)	Total, N
Sex^a^			
Female	29 (56.9)	51 (60)	80
Male	21 (41.2)	34 (40)	55
Not reported	1 (1.9)	0	1
Total	51 (100)	85 (100)	136
Discipline^b^			
Chinese medicine (Mainland China)	4 (7.8)	0 (0)	4
MBBS	5 (9.8)	8 (9.4)	13
Nursing	16 (31.4)	30 (35.3)	46
Pharmacology and pharmacy	6 (11.8)	12 (14.1)	18
Social work	5 (9.8)	5 (5.9)	10
Physiotherapy	12 (23.5)	18 (21.2)	30
Speech and hearing sciences	3 (5.9)	12 (14.1)	15
Total	51 (100)	85 (100)	136
Grade (year level)			
Year 5	1 (1.9)	0 (0)	1
Year 4	9 (17.6)	21 (24.7)	30
Year 3	15 (29.4)	20 (23.5)	35
Year 2 (half class)	14 (27.5)	16 (18.8)	30
Year 2 (new curriculum)	7 (13.7)	24 (28.2)	31
Masters year 1 (part-time)	5 (9.8)	4 (4.7)	9
Total	51 (100)	85 (100)	136

a Sex data were available for 135 participants; 1 participant did not report sex.

b All disciplines are based in Hong Kong, unless stated otherwise.

**Figure 3.**
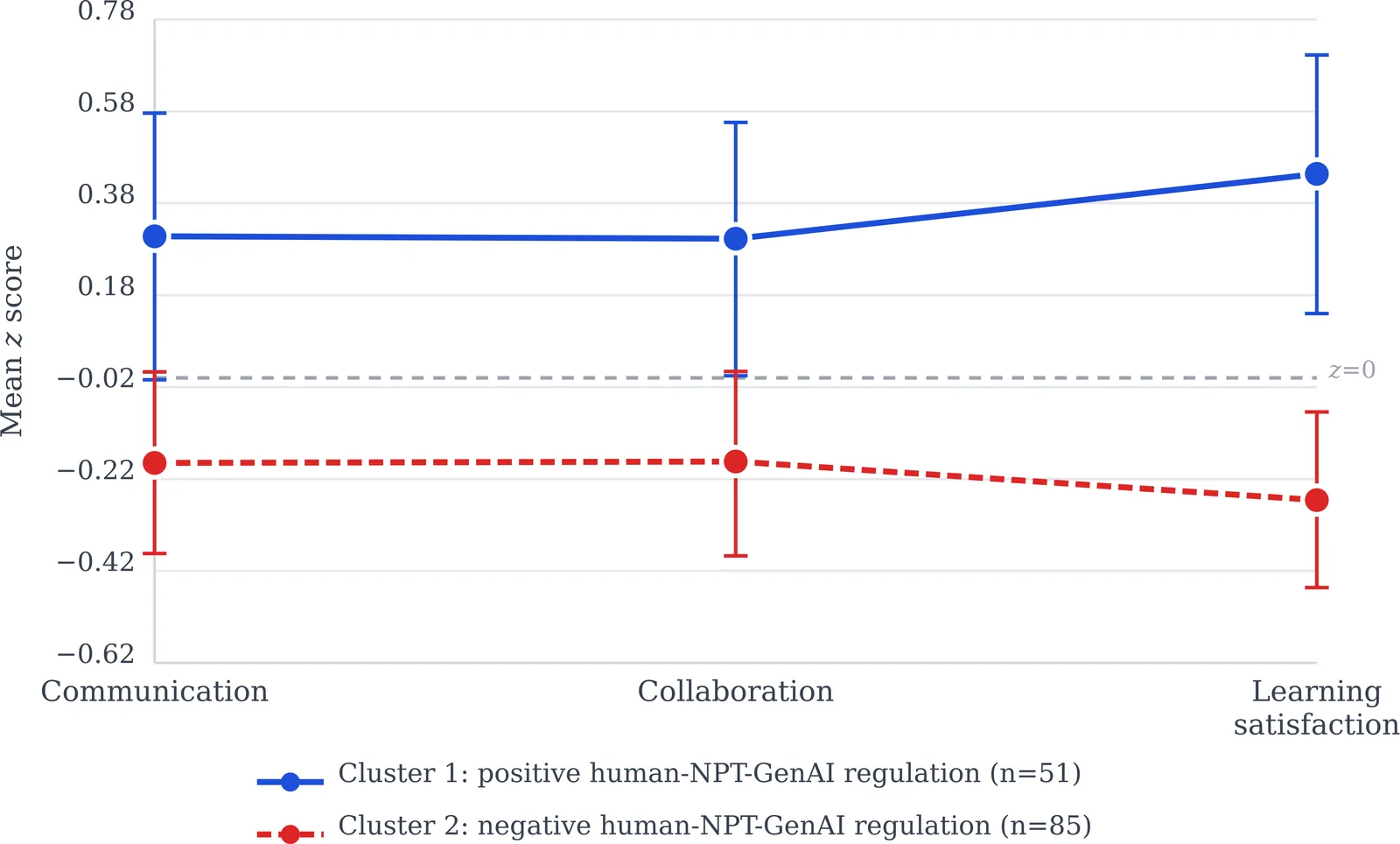
Standardized mean scores (*z* scores) for communication, collaboration, and learning satisfaction by cluster. Values are standardized *z* scores (mean 0, SD 1) computed across the full sample (N=136). Error bars represent bootstrap 95% CI based on 1000 bootstrap samples. The dashed horizontal reference line marks the overall sample mean (*z*=0). The positive human–near-peer teacher (NPT)–generative AI (GenAI) regulation cluster scored significantly higher than the negative human-NPT–GenAI regulation cluster across all 3 outcomes (see Table 5 for full statistics).

We conducted independent samples 2-tailed *t* tests to examine differences between the positive and negative human-NPT–GenAI regulation groups on 3 interprofessional learning outcomes: communication, collaboration, and learning satisfaction. Note that all means and SDs are reported as standardized (*z* score) values. Across all 3 outcomes, the positive regulation group (cluster 1) scored significantly higher than the negative regulation group (cluster 2; Table 5 and Figure 3). For communication, the positive regulation group (mean 0.31, SD 1.10) outscored the negative regulation group (mean −0.18, SD 0.89; *t*_134_=2.85; *P*=.005; mean difference [MD]=0.49, 95% CI 0.12-0.81, *d*=0.51, 95% CI 0.15-0.86). A similar pattern emerged for collaboration, with the positive regulation group (mean 0.30, SD 1.07) scoring higher than the negative regulation group (mean −0.18, SD 0.91; *t*_134_=2.81; *P*=.006; MD=0.49, 95% CI 0.13-0.83; *d*=0.50, 95% CI 0.15-0.85). The largest difference was observed for learning satisfaction, where the positive regulation group (mean 0.44, SD 1.04) substantially outscored the negative regulation group (mean −0.27, SD 0.88; *t*_134_=4.26; *P*<.001; MD=0.71, 95% CI 0.34-1.04; *d*=0.75, 95% CI 0.39-1.11). Effect sizes ranged from medium (*d*=0.50-0.51) to medium-large (*d*=0.75), and Levene test confirmed equal variances across groups for all variables (all Fs<1.00, all ps>0.30). Bootstrap 95% CI based on 1000 samples corroborated these findings. These results indicate that profile membership was associated with differences in self-reported interprofessional outcomes; however, the observational design does not allow conclusions about whether regulatory profile membership caused these differences.

**Table 5.** Independent samples 2-tailed *t* tests comparing cluster 1 (positive human-NPT–GenAI regulation) and cluster 2 (negative human-NPT–GenAI regulation) on communication, collaboration, and learning satisfaction (N=136).^a^

Variable	Cluster 1: positive human-NPT–GenAI regulation (n=51, 37.5%), mean (SD)	Cluster 2: negative human-NPT–GenAI regulation (n=85, 62.5%), mean (SD)	*t* test (*df*)	*P* value	MD^b^	Bootstrap 95% CI^c^	Cohen *d*^d^
Communication	0.31 (1.10)	−0.18 (0.89)	2.85 (134)	.005	0.49	(0.12-0.81)	0.51
Collaboration	0.30 (1.07)	−0.18 (0.91)	2.81 (134)	.006	0.49	(0.13-0.83)	0.50
Learning satisfaction	0.44 (1.04)	−0.27 (0.88)	4.26 (134)	<.001	0.71	(0.34-1.04)	0.75

a Mean and SD values are standardized (*z* scored). Equal variances were assumed for all variables (Levene test: all *F*<1.00, all *P*>.30). Outcome values are standardized *z* scores and should be interpreted as differences in self-reported outcomes, not as causal effects of profile membership.

b MD: mean difference (cluster 1-cluster 2).

c Bootstrap 95% CIs are based on 1000 bootstrap samples.

d Cohen *d* uses the pooled SD. Benchmarks: |0.2|=small, |0.5|=medium, |0.8|=large [28].

## Discussion

### Principal Findings

This person-centered study examined how health professions students reported combining SRL with perceived coregulation from NPTs and GenAI in a technology-enhanced IPE. Two perceived regulatory profiles emerged and were associated with students’ end-of-program self-reported communication, collaboration, and learning satisfaction. In interpreting these findings, it is important to emphasize that the study identifies associations among self-reported constructs rather than causal effects of NPTs or GenAI support.

### Regulatory Profiles as Distributed “Regulatory Ecologies”

Addressing RQ1, the 2-stage cluster analysis yielded a positive human-NPT–GenAI regulation profile characterized by moderately high SRL, strong coregulation from NPTs, and above-average coregulation from GenAI, as well as a negative human-NPT–GenAI regulation profile characterized by the opposite pattern. Rather than mixed configurations (eg, high SRL paired with low coregulation), we observed a “low-on-all vs high-on-all” pattern, suggesting that internal and external regulatory resources tended to accumulate rather than compensate for each other: students who regulated more effectively also reported greater perceived coregulation from human and AI supports, whereas those with weaker SRL reported limited uptake of these supports.

This accumulation pattern aligns with SRL-Coregulation perspectives that view regulation as distributed across individuals, social partners, and tools [3,13], and with person-centered work showing that adaptive resources often cluster into coherent “high-high” and “low-low” configurations [16,17]. Psychologically, the positive profile reflects a productive balance between agency and reliance: students maintain ownership of their goals and decisions yet intentionally draw on NPTs and GenAI to extend their regulatory capacity. Their use of external support is therefore not a sign of dependency but of adaptive help-seeking and willingness to distribute regulatory work when tasks are complex. NPTs and GenAI function as external “mirrors” for thinking, helping students test ideas, surface misconceptions, and maintain a coherent understanding of their team’s goals and roles. By contrast, students in the negative profile adopt a more constrained stance, combining weaker SRL with reluctance or difficulty in mobilizing available human and AI supports, leaving them more reactive and potentially more vulnerable during collaboration.

Theoretically, these findings extend SRL-coregulation perspectives by modeling NPTs and GenAI as complementary coregulatory agents within a single distributed regulatory system. Rather than treating regulation as an individual trait, the profiles illustrate how internal strategies and external supports form coherent regulatory ecologies, some richer, some poorer, with differential associations with interprofessional learning.

### Profile Differences in Interprofessional Outcomes

Addressing RQ2, students in the positive profile reported significantly higher interprofessional communication and collaboration, and greater learning satisfaction than those in the negative profile, with effect sizes ranging from medium (communication and collaboration, *d*=0.50-0.51) to medium-large (satisfaction, *d*=0.75). These findings support H2.1 and H2.2 and suggest that students’ self-regulatory and coregulatory configurations are associated with educationally meaningful differences in self-reported outcomes.

The advantage of the positive profile is consistent with evidence that higher SRL is associated with better performance and readiness for lifelong learning in health professions education [5-7] and that high-quality instructional and social supports are associated with collaborative processes and satisfaction [12,29]. Our findings add nuance by showing that it is the combination of stronger SRL with active engagement with NPT and GenAI supports, a richer regulatory ecology, that is associated with more favorable interprofessional outcomes, rather than any single resource in isolation.

Equally important are the weaker outcomes for the negative profile. These students entered with lower SRL and reported limited engagement with both NPT and GenAI coregulation, despite having equivalent access to these supports. Their lower communication, collaboration, and satisfaction echo work showing that learners with weaker SRL and less use of external supports are at risk in complex, self-directed tasks [8]. For IPE specifically, this suggests that regulatory vulnerabilities may undermine the development of collaborative competence even when institutional resources are available.

The pattern for GenAI deserves particular attention. Students in the positive profile reported stronger perceived coregulation from GenAI across planning, conceptual clarification, monitoring, and reflection, and this was associated with better outcomes. This offers early empirical support for the idea that large language models may be perceived by students as coregulatory partners when students engage with them productively [9,15]. At the same time, the sizable negative profile indicates that simply providing access to GenAI and NPT facilitators is insufficient; students with weaker SRL may not spontaneously perceive or use these supports as regulatory resources.

### Implications for Designing Human-AI Coregulation in and Beyond IPE

Theoretically, the findings support a distributed view of regulation in which learners’ SRL is situated within a broader ecology of human and technological supports. In this ecology, NPTs and GenAI may serve different but complementary regulatory functions. NPTs can provide socially responsive scaffolding, monitor group interaction, prompt participation across professions, and support emotional or motivational regulation. GenAI, by contrast, can provide on-demand prompts, alternative explanations, planning support, and opportunities for reflection. The value of a human-AI regulatory ecology may therefore lie not in replacing human facilitation with AI but in coordinating the strengths of both forms of support.

Practically, SRL and coregulation should be treated as explicit design targets in technology-enhanced IPE. Educators should design NPTs and GenAI supports as explicit regulatory scaffolds, rather than assuming students will use them productively on their own. NPTs and GenAI can be positioned as “regulation partners” whose roles are aligned with key SRL processes. For example, near-peers can guide planning and monitoring, while GenAI prompts can be structured to support checking assumptions, exploring alternative management plans, and promoting reflective practice. Making these roles explicit in instructions and debriefings may help students, especially those in the negative profile, use available supports more effectively.

The implications also extend beyond IPE. In project-based learning, team-based Science, Technology, Engineering, and Mathematics courses, teacher education, clinical simulation, and online collaborative learning, students increasingly work with both human facilitators and AI tools. The present findings suggest that simply providing access to these supports may be insufficient, particularly for learners who report weaker SRL. Learners may need explicit instruction in how to seek help, evaluate AI responses, use prompts strategically, and integrate feedback from human and AI sources. Thus, human-AI learning environments should include not only access to technology but also regulatory routines, prompt guidance, reflection templates, and opportunities for facilitated debriefing.

The identification of a vulnerable negative profile also suggests potential opportunities for targeted intervention. These students may benefit from early SRL workshops (eg, on goal setting, strategic planning, and structured reflection) combined with more structured requirements to engage with near-peers and GenAI (eg, mandatory planning templates cocompleted with NPTs, guided GenAI prompts embedded in cases). Conversely, students in the positive profile may require less basic scaffolding and could be challenged to take on leadership roles or to critically interrogate GenAI suggestions.

Brief profile screening early in an IPE program, using short SRL questionnaires and early perceived-support or help-seeking questionnaires, could help educators identify students who may be at risk of remaining in a low-regulation, low-support pattern. In resource-constrained settings, such information could guide the allocation of mentoring and technological resources to those most likely to benefit.

### Limitations and Future Directions

Several limitations should be noted. First, the modest sample size (N=136) may limit statistical power, the stability of the person-centered profiles, and transferability to other IPE contexts. Although the 2-profile solution was interpretable and supported by cluster diagnostics, person-centered results can be sensitive to sample composition and analytic decisions. Second, all focal constructs were assessed via self-report. This is a substantive limitation because SRL, coregulation from NPTs, coregulation from GenAI, communication, collaboration, and satisfaction were all reported by the same respondents. As a result, the observed associations may partly reflect shared method variance, general positive response tendencies, or students’ overall satisfaction with the program rather than distinct regulatory processes. The very high correlation between communication and collaboration also suggests that students may not have clearly differentiated these competencies in their self-assessments. Future work should incorporate observational data, peer and NPT ratings, performance-based assessments, video-coded team interaction, and learning analytics to capture coregulation processes more directly. Third, GenAI use was self-selected and was not objectively tracked. We did not record which tools students used, how often they used them, or the content and quality of their prompts and GenAI-generated responses. Therefore, low GenAI coregulation scores may reflect nonuse, limited access, weaker prompting, or low perceived usefulness, and the findings cannot be attributed to any particular GenAI platform. Fourth, the design was not experimental, and the person-centered analysis involved analytic choices (eg, number of clusters) that may influence results. Experimental or quasi-experimental designs that manipulate the availability or structure of near-peer and GenAI support, combined with longitudinal profiling, would provide stronger evidence about how regulatory ecologies develop and can be reshaped.

### Conclusions

This study suggests that health professions students in a technology-enhanced IPE program formed distinct perceived regulatory configurations, differing jointly in SRL and coregulation from NPTs and GenAI, and that these configurations were associated with different interprofessional outcomes. These findings suggest that students’ perceived regulatory ecologies are associated with self-reported interprofessional learning outcomes. For educators, the key message is that NPT facilitation and GenAI tools should be designed as coordinated regulatory supports rather than offered as isolated enhancements, with particular attention to scaffolding students who do not spontaneously engage with these resources. For researchers, the findings point to the value of person-centered, multimethod, and longitudinal designs that trace how human-AI regulatory ecologies evolve and how they can be reshaped to foster more adaptive self-regulation and coregulation in collaborative professional learning.

## Abbreviations

GenAI: generative AI
GFI: goodness of fit index
IFI: incremental fit index
IPE: interprofessional education
MD: mean difference
MSLQ: Motivated Strategies for Learning Questionnaire
NFI: normed fit index
NPT: near-peer teacher
RMSEA: root mean square error of approximation
SRL: self-regulated learning
SRMR: standardized root mean square residual
